# Supplementary material for: Impacts of complex behavioral responses on asymmetric interacting spreading dynamics in multiplex networks
Source: Sci Rep. 2016 May 9;6:25617. doi: 10.1038/srep25617 (PMC4860576; doi:10.1038/srep25617)
Supplement: Supplementary Information [file srep25617-s1.pdf]

Supporting Information for

Impacts of complex behavioral responses on asymmetric interacting spreading dynamics in multiplex networks

Quan-Hui Liu, Wei Wang, Ming Tang and Hai-Feng Zhang

S1. Theoretical analysis

The heterogeneous mean-field theory [1] was adopted to derive the mean-field equations for the uncorrelated double-layer network. Let $P_A(k_A)$ $[P_B(k_B)]$ be the degree distribution of communication layer A (contact layer B), and the average degrees of A and B are $\langle k_A\rangle = \sum_{k_A} k_A P_A(k_A)$ and $\langle k_B\rangle = \sum_{k_B} k_B P_B(k_B)$, respectively. Meanwhile, we assume that inner-layer links and inter-layer links have no degree correlations. The variables of $s^A_{k_A}(t)$, $\rho^A_{k_A}(t)$ and $r^A_{k_A}(t)$ are used to denote the densities of the susceptible, informed, and recovered nodes with degree k_A in layer A at time t, respectively. Thereinto, $\rho^A_{k_A}(t) = \sum_m \rho^A_{k_A}(m,t)$, and $\rho^A_{k_A}(m,t)$ is the density of I_A nodes with degree k_A who has received m pieces of information till time t. Similarly, $s^B_{k_B}(t)$, $\rho^B_{k_B}(t)$, $r^B_{k_B}(t)$ and $v^B_{k_B}(t)$ are the densities of the susceptible, infected, recovered and vaccinated nodes with degree k_B in layer B at time t, respectively.

A. Mean-field rate equations

The mean-field rate equation of the information spreading in layer A is

$$\frac{ds^A_{k_A}(t)}{dt} = -s^A_{k_A}(t)[\Psi^A_{S_A,k_A}(t) + \sum_{k_B} P_B(k_B)\Psi^B_{S_B,k_B}(t)], \tag{S1}$$

For $m = 1$, the rate equation of $\rho^A_{k_A}(1,t)$ is given as

$$\frac{d\rho^A_{k_A}(1,t)}{dt} = s^A_{k_A}(t)\sum_{n=1}^{k_A}\pi^A_{S_A,k_A}(n)B_{n,1}(\beta_A) + s^A_{k_A}(t)\sum_{k_B} P_B(k_B)\Psi^B_{S_B,k_B}(t) - \rho^A_{k_A}(1,t)\Psi^A_{I_A,k_A}(t) - \mu\rho^A_{k_A}(1,t), \tag{S2}$$

When $m > 1$, the rate equation of $\rho^A_{k_A}(m,t)$ is described as

$$\frac{d\rho^A_{k_A}(m,t)}{dt} = s^A_{k_A}(t)\sum_{n=m}^{k_A}\pi^A_{S_A,k_A}(n)B_{n,m}(\beta_A) + \sum_{q=1}^{m-1}\rho^A_{k_A}(q,t)\sum_{n=m-q}^{k_A}\pi^A_{I_A,k_A}(n)B_{n,m-q}(\beta_A) - \rho^A_{k_A}(m,t)\Psi^A_{I_A,k_A}(t) - \mu\rho^A_{k_A}(m,t), \tag{S3}$$

$$\frac{dr^A_{k_A}(t)}{dt} = \mu\sum_m \rho^A_{k_A}(m,t). \tag{S4}$$

The mean-field rate equation of the epidemic spreading in layer B is

$$\frac{ds^B_{k_B}(t)}{dt} = -s^B_{k_B}(t)\Psi^B_{S_B,k_B}(t) - \sum_{k_A}\chi^A_{S_A,k_A}(t) - s^B_{k_B}(t)\sum_{k_A}\chi^A_{I_A,k_A}(t), \tag{S5}$$

$$\frac{d\rho^B_{k_B}(t)}{dt} = s^B_{k_B}(t)\Psi^B_{S_B,k_B}(t) - \mu\rho^B_{k_B}(t), \tag{S6}$$

$$\frac{r^B_{k_B}(t)}{dt} = \mu\rho^B_{k_B}(t), \tag{S7}$$

$$\frac{dv_{k_B}^B(t)}{dt} = \sum_{k_A} \chi_{S_A,k_A}^A(t) + s_{k_B}^B(t) \sum_{k_A} \chi_{I_A,k_A}^A(t). \tag{S8}$$

From Eqs. (S1)-(S8), the density associated with each distinct state in layer A or B is given by

$$x^H(t) = \sum_{k_H=1}^{k_H,max} P_H(k_H) x_{k_H}^H(t),$$

where $H \in \{A, B\}$, $x \in \{s, \rho, r, v\}$, and $k_{H,min}$ ($k_{H,max}$) denotes the smallest (largest) degree of layer H. Specially, the density of I_A node with degree k_A in layer A is $\rho_{k_A}^A(t) = \sum_m \rho_{k_A}^A(m,t)$. The final densities of the whole system can be obtained by taking the limit $t \to \infty$.

B. Linear analysis of information threshold in layer A

On an uncorrelated nonoverlapping double-layer network, at the outset of the spreading dynamics, the whole system can be regarded as consisting of two coupled SI-epidemic subsystems [2] with the time evolution described by equations (S2),(S3) and (S6). As $t \to 0$, one has $s_{k_A}^A(t) \approx 1$ and $s_{k_B}^B(t) \approx 1$, which reduce equations (S2),(S3) and (S6) as

$$\begin{cases} \frac{d\rho_{k_A}^A(1,t)}{dt} = \beta_A k_A \Theta_{S_A}^A(t) + \beta_B \langle k_B \rangle \Theta_{S_B}^B(t) - \mu \rho_{k_A}^A(1,t), \\ \frac{d\rho_{k_A}^A(m,t)}{dt} = 0 \; (m > 1), \\ \frac{d\rho_{k_B}^B(t)}{dt} = \beta_B k_B \Theta_{S_B}^B(t) - \mu \rho_{k_B}^B(t). \end{cases} \tag{S9}$$

The above equations can be simplified as matrix form:

$$\frac{d\vec{\rho}}{dt} = \frac{C\vec{\rho}}{\mu} - \vec{\rho}, \tag{S10}$$

where

$$\vec{\rho} \equiv (\rho_{k_A=1}^A(1), \ldots, \rho_{k_{A,max}}^A(1), \rho_{k_B=1}^B, \ldots, \rho_{k_{B,max}}^B)^T. \tag{S11}$$

The matrix C is written as a block matrix:

$$C = \begin{pmatrix} C^A & D^B \\ 0 & C^B \end{pmatrix}, \tag{S12}$$

whose elements are given as

$$\begin{aligned} C_{k_A,k'_A}^A &= [\beta_A k_A (k'_A - 1) P_A(k'_A)]/\langle k_A \rangle, \\ C_{k_B,k'_B}^B &= [\beta_B k_B (k'_B - 1) P_B(k'_B)]/\langle k_B \rangle, \\ D_{k_B,k'_B}^B &= \beta_B (k'_B - 1) P_B(k'_B). \end{aligned}$$

In general, information spreading in layer A can be facilitated by the outbreak of the epidemic in layer B, since an infected node in layer B instantaneously makes its counterpart node in layer A "infected" by the information immediately and certainly. That is to say, the number of the informed nodes in layer A is larger than the number of the infected nodes in layer B. If the maximum eigenvalue Λ_C of matrix C/μ is greater than 1, an outbreak of the information will occur absolutely [3]. We then have

$$\Lambda_C = \max\{\Lambda_A, \Lambda_B\}, \tag{S13}$$

where max $\{\}$ denotes the greater of the two, and

$$\begin{aligned} \Lambda_A = \beta_A(\langle {k_A}^2 \rangle - \langle k_A \rangle)/(\mu \langle k_A \rangle), \\ \Lambda_B = \beta_B(\langle {k_B}^2 \rangle - \langle k_B \rangle)/(\mu \langle k_B \rangle), \end{aligned}$$

are the maximum eigenvalues of matrices C_A and C_B [4], respectively. Thus, the outbreak threshold for the spreading in layer A is given as

$$\beta_{Ac} = \begin{cases} \beta_{Au}, & for \quad \beta_B \le \beta_{Bu}; \\ 0, & for \quad \beta_B > \beta_{Bu}. \end{cases} \tag{S14}$$

Here $\beta_{Au} \equiv \mu\langle k_A\rangle/(\langle {k_A}^2\rangle - \langle k_A\rangle)$ and $\beta_{Bu} \equiv \mu\langle k_B\rangle/(\langle {k_B}^2\rangle - \langle k_B\rangle)$ denote the outbreak threshold of information spreading in layer A when it is isolated from layer B, and the outbreak threshold of epidemic spreading in layer B when the coupling between the two layers is absent, respectively.

C. Competing percolation theory for epidemic threshold in layer B

For $\beta_A < \beta_{Au}$, Eq. (S14) shows that the information cannot break out in layer A if layer A and layer B are isolated. When the two spreading dynamics are interacting, *near the epidemic threshold*, the spread of epidemic in layer B can only lead to a few of counterpart nodes in layer A "infected" with the information, and thus these informed nodes in layer A have negligible effect on the epidemic dynamics in layer B since $\beta_A < \beta_{Au}$. The above explanation indicates that $\beta_{Bc} \approx \beta_{Bu}$ when $\beta_A < \beta_{Au}$. However, for $\beta_A > \beta_{Au}$, the information outbreak in layer A which makes many counterpart nodes in layer B vaccinated, thus hinders the spread of epidemic in layer B. Once a node is in the vaccination state, it will no longer be infected. Usually, we can regard this kind of vaccination as a type of "disease," and every node in layer B can be in one of the two states: infected or vaccinated. Epidemic spreading and vaccination diffusion (derived by information diffusion) can thus be viewed as a pair of competing "diseases" spreading in layer B [5]. As pointed out by Karrer and Newman [5], in the limit of large network size N and the two competing diseases with different growth rates, then they can be treated as if they were in fact spreading non-concurrently, one after the other.

To clarify the interplay between epidemic and vaccination spreading, we should determine which one is the faster "disease". At the early stage, the average number of infected nodes in the isolated layer B grows exponentially as $N_e(t) = n_0(R_e)^t = n_0e^{t\ln R_e}$, where $R_e = \beta_B/\beta_{Bu}$ is the basic reproductive number for the disease in the isolated layer B [2], and n_0 denotes the number of initially infected nodes. Similarly, for information spreading in the isolated layer A, the average number of informed nodes at the early time is $N_i(t) = n_1(R_i)^t = n_1e^{t\ln(R_i)} = N\sum_m \rho^A(m,t)$, where $n_0 = n_1$, $\rho^A(m,t) = \sum_{k_A} P_A(k_A)\rho^A_{k_A}(m,t)$ denotes the density of the nodes who have received m pieces of information till time step t, and $R_i = \beta_A/\beta_{Au}$ is the reproductive number for information spreading in the isolated layer A. So the number of vaccination nodes is $N_V(t) = N\sum_m \rho^A(m,t)\xi_m$, which is larger than $\xi_1 n_0 e^{t\ln(R_i)}$ since $\xi_m > \xi_1$, and which is smaller than $n_0e^{t\ln(R_i)}$ since $\xi_m < 1$. As a result, at the early stage, we can view that N_v grows exponentially and the growth satisfies $N_V \sim O(N_i)$.

Since the number of vaccination and infection both grow in an exponentially way, we can obtain the ratio of their growth rates as

$$\theta = \frac{R_i}{R_e} = \frac{\beta_A\beta_{Bu}}{\beta_B\beta_{Au}}. \tag{S15}$$

When $\theta < 1$, *i.e.*, $\beta_B\beta_{Au} > \beta_A\beta_{Bu}$, the disease process grows faster than the vaccination process. In this case, the effect of vaccination is insignificant and can be neglected. However, when $\theta > 1$, the information process spreads faster than the epidemic process, which is in accord with realistic situations since many on-line social networks and mass media can promote the spreading of information. Given that vaccination and epidemic can be treated successively and separately, by letting $\beta_B = 0$ and obtaining the final density of vaccination $v^B(\infty)|_{\beta_B=0}$ from Eq. (S8), the threshold of epidemic outbreak is given as [6]

$$\beta_{Bc} = \frac{\mu\langle k_B\rangle}{[1 - v^B(\infty)|_{\beta_B=0}](\langle {k_B}^2\rangle - \langle k_B\rangle)}. \tag{S16}$$

S2. Simulation results

We first describe the simulation processes of the two spreading dynamics in double-layer networks, and then present results for RR-ER double-layer and SF-SF double-layer networks. Lastly, we study the effect of different relative cost of vaccination and treatment on total social cost in SF-ER double layer networks.

A. Simulation process

To initiate an epidemic spreading process, a node in layer B is randomly infected and its counterpart node in layer A is thus in the informed state, too. The updating process is performed with parallel dynamics, which is widely used in statistical physics [7]. At each time step, we first calculate the informed (infected) probability $\pi_A = 1 - (1 - \beta_A)^{n_I^A}$ [$\pi_B = 1 - (1 - \beta_B)^{n_I^B}$] that

each susceptible or informed node in layer A may be informed or informed again by its informed neighbors and each susceptible node in layer B infected by its infected neighbors, where n_I^A (n_I^B) is the number of its informed (infected) neighboring nodes.

According to the dynamical mechanism, once node A_i is in the susceptible state, its counterpart node B_i will be also in the susceptible state. Besides, when a node in layer A is in the informed state, its counterpart node may be in the susceptible state. Considering the asymmetric coupling between the two layers in these two cases, both the information-transmission and disease-transmission events can hardly occur at the same time. Thus, with probability $\pi_A/(\pi_A + \pi_B)$, node A_i have a probability π_A to get the information from its informed neighbors in layer A. If node A_i is informed, its counterpart node B_i will turn into the vaccination state with probability ξ_m, where m is total times of information the node has received. With probability $\pi_B/(\pi_A + \pi_B)$, node B_i have a probability π_B to get the infection from its infected neighbors in layer B, and then node A_i also get the information about the disease.

In the other case that node B_i and its corresponding node A_i are in the susceptible state and the informed (or refractory) state respectively, only the disease-transmission event can occur at the time step. Thus, node B_i will be infected with probability π_B.

After renewing the states of susceptible nodes, each informed (infected) node can enter the recovering phase with probability $\mu = 0.5$. The spreading dynamics terminates when all informed (or infected) nodes in both layers are recovered, and the final densities r^A, r^B, and v^B are then recorded. The simulations are implemented using 30 different double-layer network realizations and each realization is repeated 2×10^3 times. The network size of $N_A = N_B = 1 \times 10^4$ and average degrees $\langle k_A \rangle = \langle k_B \rangle = 8$ are used for all subsequent numerical results, unless otherwise specified.

B. RR-ER double-layer network

In RR-ER double-layer network, we also investigate the impacts of social reinforcement effect on the two types of spreading dynamics. At first, We use the standard configuration model [9] to generate regular random network (RR) for the communication subnetwork (layer A). The contact subnetwork in layer B is of the Erdős and Rényi (ER) type [8]. We use the notation RR-ER to denote the double-layer network. The sizes of both layers are set to be $N_A = N_B = 1 \times 10^4$ and their average degrees are $\langle k_A \rangle = \langle k_B \rangle = 8$. And we set $\xi_1 = 0.05$, $\mu = 0.5$ in the following simulations. As shown in Figs. S1, S2, S3 and S4, we obtain the similar results of social reinforcement effect on the two types of spreading dynamics as in SF-ER double network.

C. SF-SF double-layer network

In SF-SF double-layer network, we also investigate the impacts of social reinforcement effect on the two types of spreading dynamics. At first, We use the standard configuration model to generate networks with power-law degree distributions [9–11] for the communication subnetwork (layer A), with $P_A(k_A) = \zeta k_A^{-\gamma_A}$, $\zeta = 1/\sum_{k_{min}}^{k_{max}} k_A^{-\gamma_A}$, $\gamma = 3.0$ and the maximum degree $k_{max} \sim N^{1/(\gamma_A - 1)}$. The contact subnetwork in layer B is generated with the same methods as layer A. We use the notation SF-SF to denote the double-layer network. The sizes of both layers are set to be $N_A = N_B = 1 \times 10^4$ and their average degrees are $\langle k_A \rangle = \langle k_B \rangle = 8$. And we set $\xi_1 = 0.05$, $\mu = 0.5$ in the following simulations. As shown in Figs. S5, S6, S7 and S8, we obtain the similar results of social reinforcement effect on the two types of spreading dynamics as in SF-ER double network.

D. Different relative cost of vaccination and treatment

We study the different relative costs of vaccination and treatment to the effect of optimal control in SF-ER double-layer networks. we have assumed that the cost of treatment is twice and five times of vaccination cost, as shown in Fig. S9 and Fig. S10, respectively. We find when the information spreads faster than the disease, there still exists an optimal α yielding the least social cost. When the information about disease spreads slowly, increasing α can result in less social cost. These results have shown that the different relative costs of vaccination and treatment do not influence previous conclusion qualitatively.

---

[1] Barthélemy, M., Barrat, A., Pastor-Satorras, R. & Vespignani, A. Velocity and Hierarchical Spread of Epidemic Outbreaks in Scale-Free Networks. *Phys. Rev. Lett.* **92**, 178701 (2004).

[2] Newman, M. E. J. *Networks An Introduction* (Oxford University Press, Oxford, 2010).

[3] Saumell-Mendiola, A., Serrano, M. Á. & Boguñá, M. Epidemic spreading on interconnected networks. *Phys. Rev. E* **86**, 026106 (2012).

[4] Mieghem, P. V. *Graph Spectra for Complex Networks* (Cambrige university press, England, 2011).

[5] Karrer, B. & Newman, M. E. J. Competing epidemics on complex networks. *Phys. Rev. E* **84**, 036106 (2011).

[6] Wang, W. *et al.* Asymmetrically interacting spreading dynamics on complex layered networks. *Sci. Rep.* **4**, 5097 (2014).

[7] Marro, J. & Dickman, R. *Nonequilibrium Phase Transitions in Lattice Models* (Cambrige university press, Cambrige, 1999).

[8] Erdős, P. & Rényi. On random graphs. *Publ. Math.* **6**, 290-297 (1959).

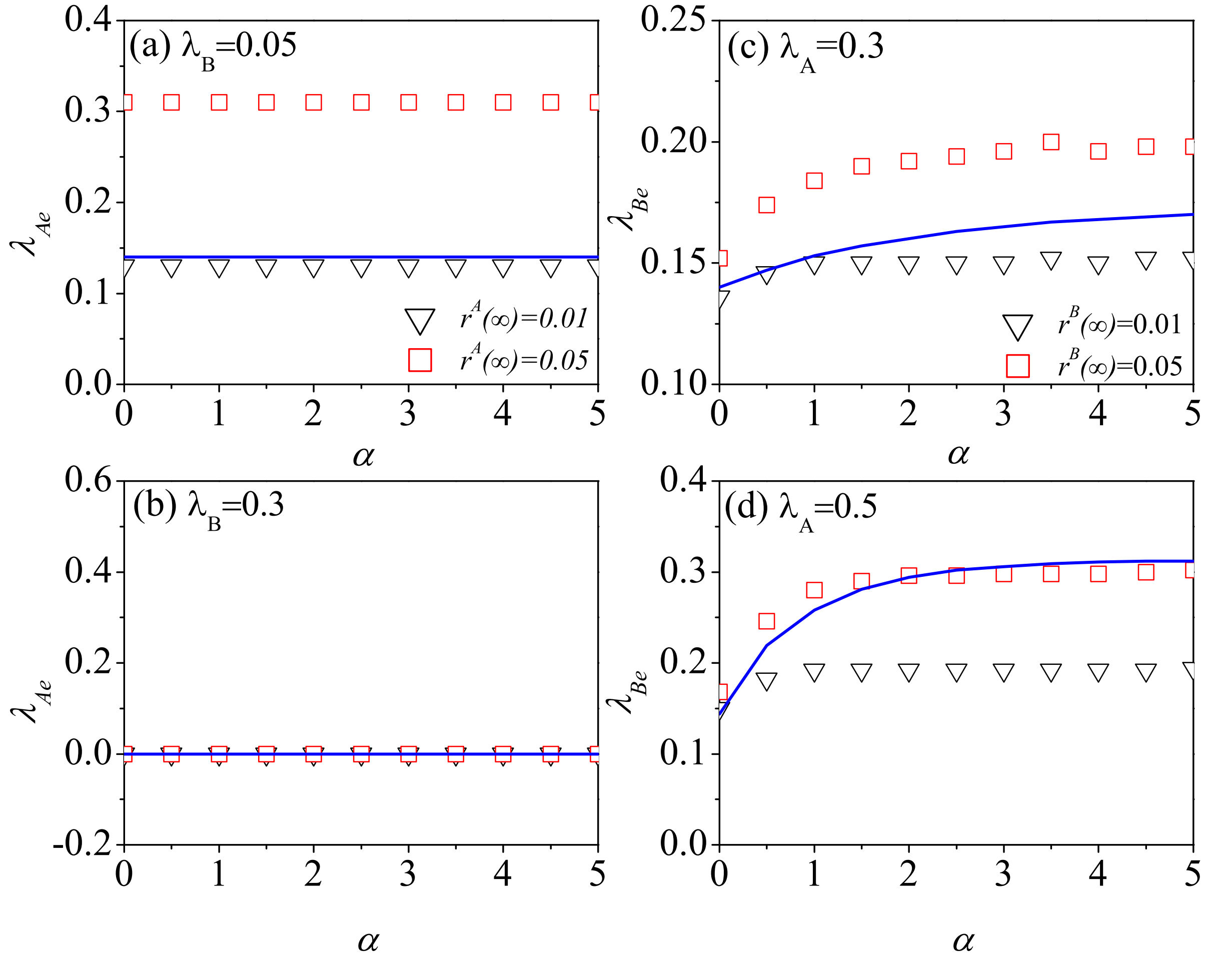

FIG. S1: **The impacts of social reinforcement effect on the outbreak threshold.** For RR-ER double-layer network, the reference information threshold λ_{Ae} and the reference epidemic threshold λ_{Be} as the function of α are obtained by numerical simulation. Owing to the difficulty of determining the threshold values from numerical predictions, we respectively take the critical density where the final recovery density in layer A (B) are 0.01 (gray circles), 0.02 (oliver downtriangles) and 0.05(blue squares) as the reference threshold values. The red solid line is the corresponding theoretical prediction from Eqs. (S14) and (S16). (a) In communication layer A, the reference information threshold λ_{Ae} as a function of α when λ_B is set as 0.5; (b) In physical contact layer B, the reference epidemic threshold λ_{Be} as a function of α at $\lambda_A = 0.5$.

[9] Newman, M. E. J., Strogatz, S. H. & Watts, D. J. Random graphs with arbitrary degree distributions and their applications. *Phys. Rev. E* **64**, 026118 (2001).
[10] Newman, M. E. J. Power laws, Pareto distributions and Zipf's law. *Contemp. Phys.* **46**, 323-351 (2005).
[11] Catanzaro, M., Boguñá, M. & Pastor-Satorras, R. Generation of uncorrelated random scale-free networks. *Phys. Rev. E* **71**, 027103 (2005).

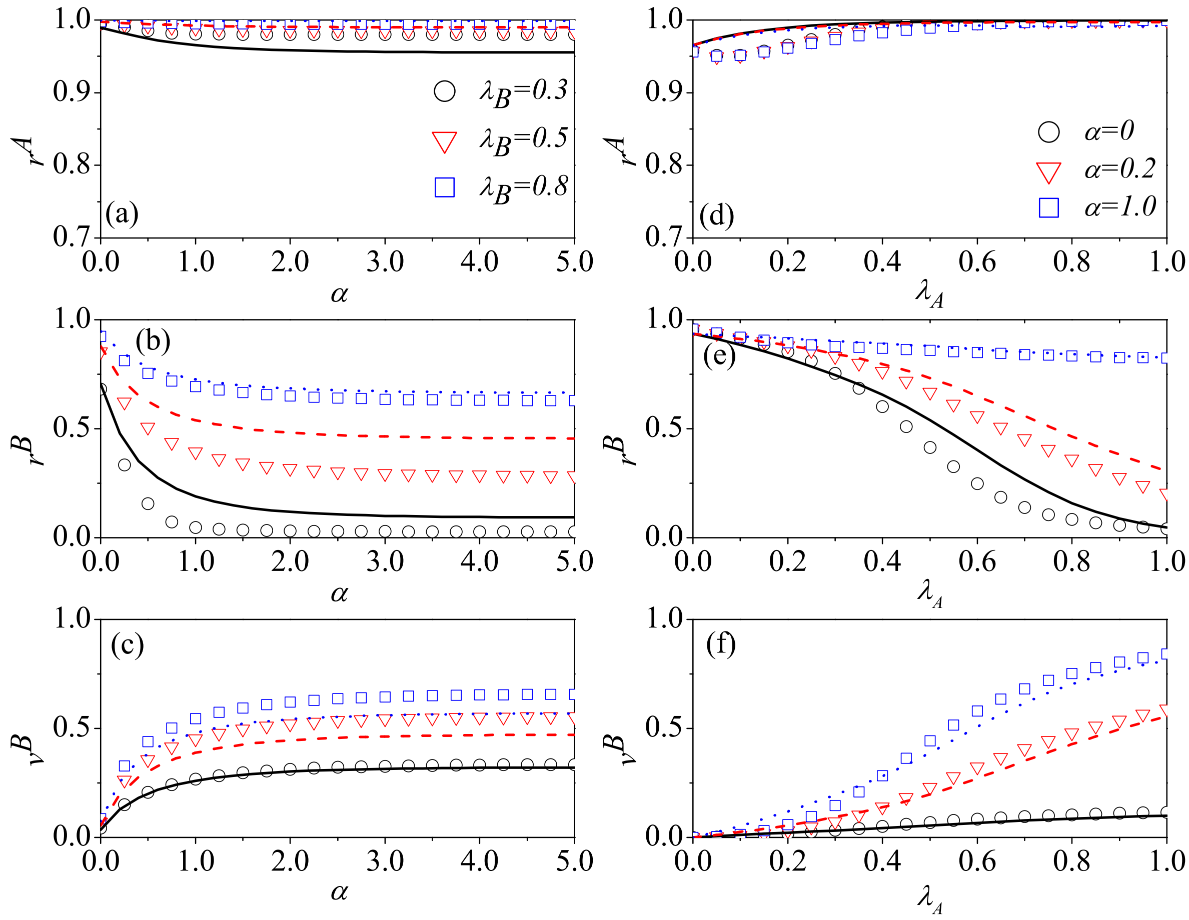

FIG. S2: **The impacts of social reinforcement effect and information transmission rate on final states.** For RR-ER double-layer network, subfigures (a), (b), and (c) show the values of r^A, r^B and v^B as a function of α for different values of λ_B (0.3, 0.5, and 0.8), with the analytical predictions corresponding to the black solid, red dashed, and blue doted lines, respectively. When λ_A is set as 0.5. Subfigures (d), (e), and (f) illustrate the values of r^A, r^B and v^B versus the parameter λ_A for different values of α (0, 0.2, and 1.0), corresponding to the black solid, red dashed, and blue doted lines respectively. When λ_B is fixed at 0.5.

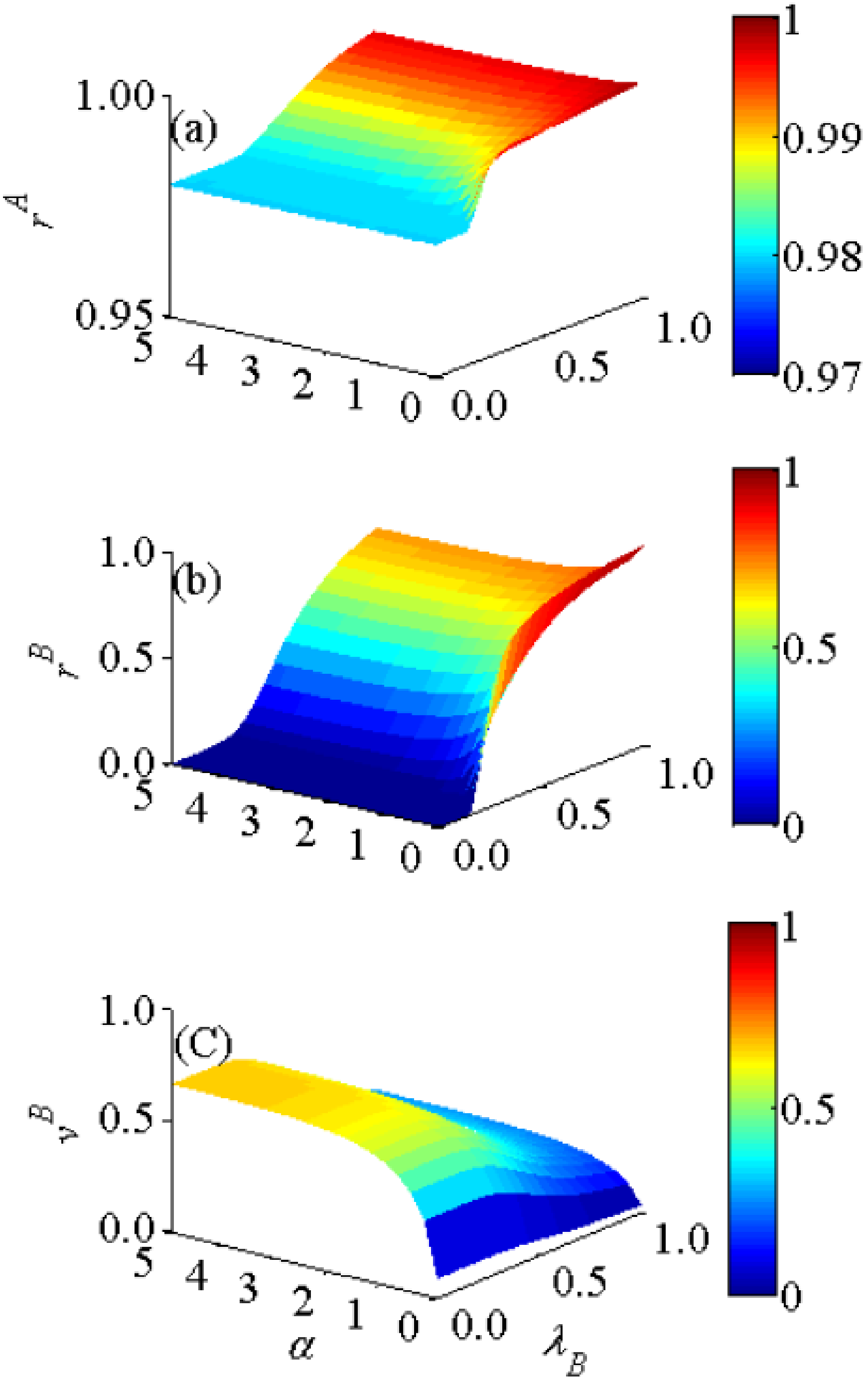

FIG. S3: **A systematic investigation of social reinforcement effect and disease transmission rate impact on final states.** For RR-ER double-layer network, (a) recovered density r^A, (b) recovered density r^B, (c) the vaccination density v^B versus α and β_B for $\lambda_A = 0.5$.

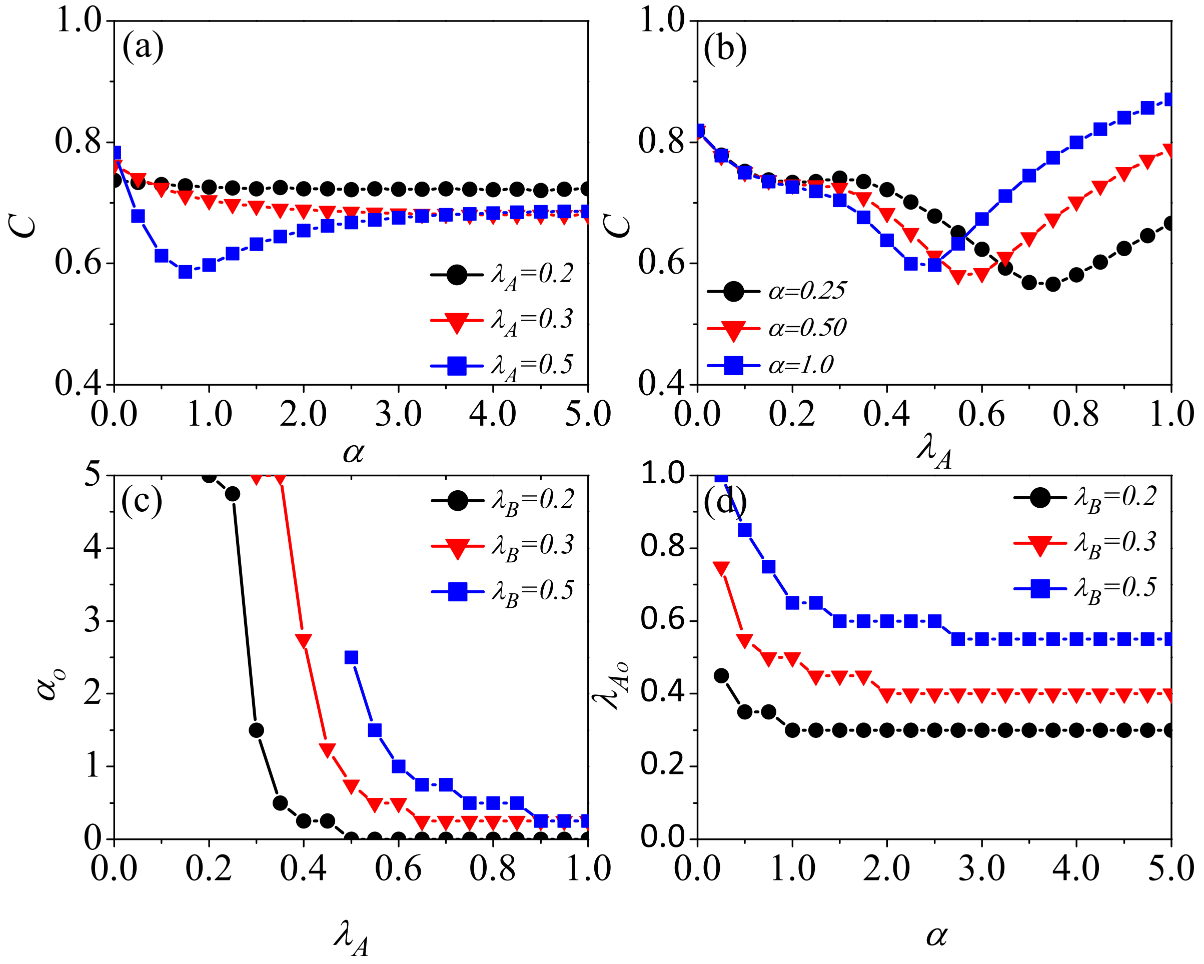

FIG. S4: **Impacts of social reinforcement effect and information transmission rate on the social cost and the optimal control.** For RR-ER double-layer network, the social cost C versus the parameters of α and λ_A in subfigures (a) and (b), respectively. Here the value of λ_B is fixed at 0.3. The optimal α_o versus β_A and optimal λ_{Ao} versus α in subfigures (c) and (d), respectively. In (a), we select three different values of λ_A(0.2, 0.3, and 0.5), corresponding to the black circle solid, red triangle solid, and blue square solid lines, respectively. In (b), different values of α (0.25, 0.5 and 1.0) corresponds to the black circle solid, red triangle solid, and blue square solid lines, respectively. (c) the α_o versus λ_A and (d) the λ_{Ao} versus α under different λ_B (0.2, 0.3 and 0.5) corresponds to the black circle solid, red triangle solid, and blue square solid lines, respectively.

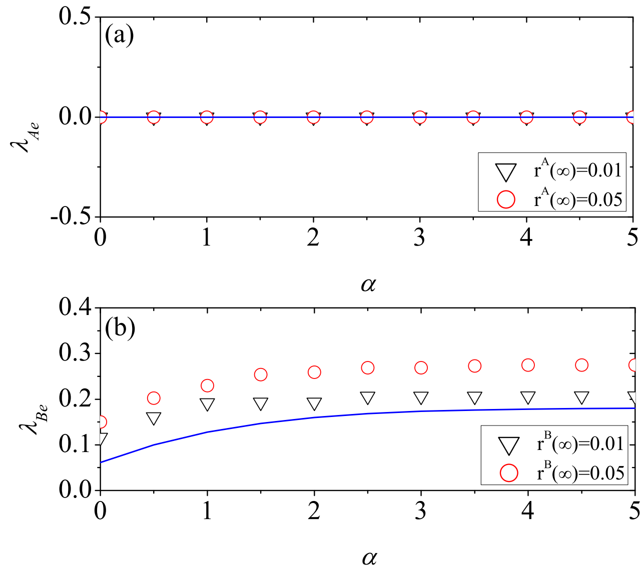

FIG. S5: **The impacts of social reinforcement effect on the outbreak threshold.** For SF-SF double-layer network, the reference information threshold λ_{Ae} and the reference epidemic threshold λ_{Be} as the function of α are obtained by numerical simulation. Owing to the difficulty of determining the threshold values from numerical predictions, we respectively take the critical density where the final recovery density in layer A (B) are 0.01 (black down triangles), and 0.05(red circles) as the reference threshold values. The blue solid line is the corresponding theoretical prediction from Eqs. (S14) and (S16). (a) In communication layer A, the reference information threshold λ_{Ae} as a function of α when λ_B is set as 0.5; (b) In physical contact layer B, the reference epidemic threshold λ_{Be} as a function of α at $\lambda_A = 0.5$.

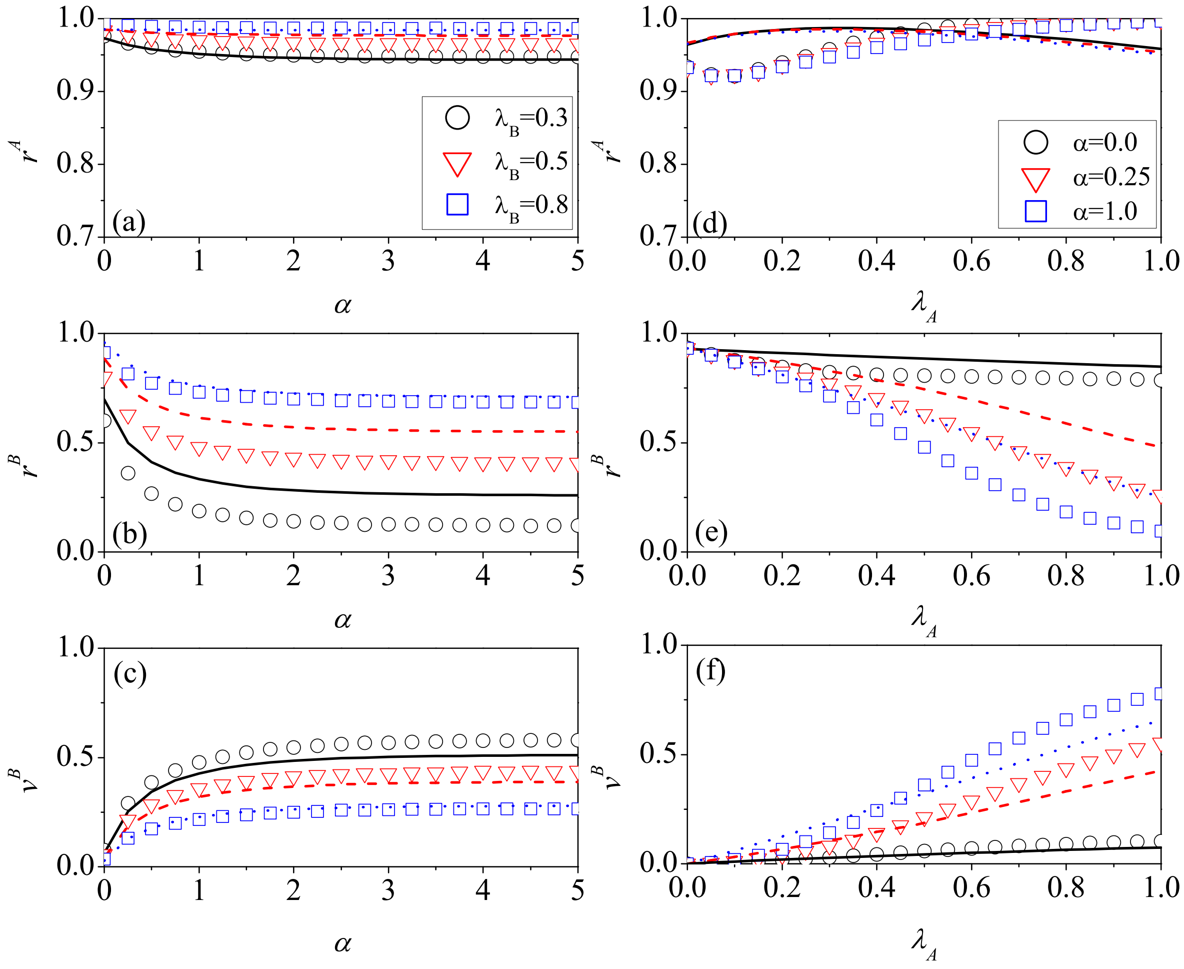

FIG. S6: **The impacts of social reinforcement effect and information transmission rate on final states.** For SF-SF double-layer network, subfigures (a), (b), and (c) show the values of r^A, r^B and v^B as a function of α for different values of λ_B (0.3, 0.5, and 0.8), with the analytical predictions corresponding to the black solid, red dashed, and blue doted lines, respectively. When λ_A is set as 0.5. Subfigures (d), (e), and (f) illustrate the values of r^A, r^B and v^B versus the parameter λ_A for different values of α (0.0, 0.25, and 1.0), corresponding to the black solid, red dashed, and blue doted lines respectively. When λ_B is fixed at 0.5.

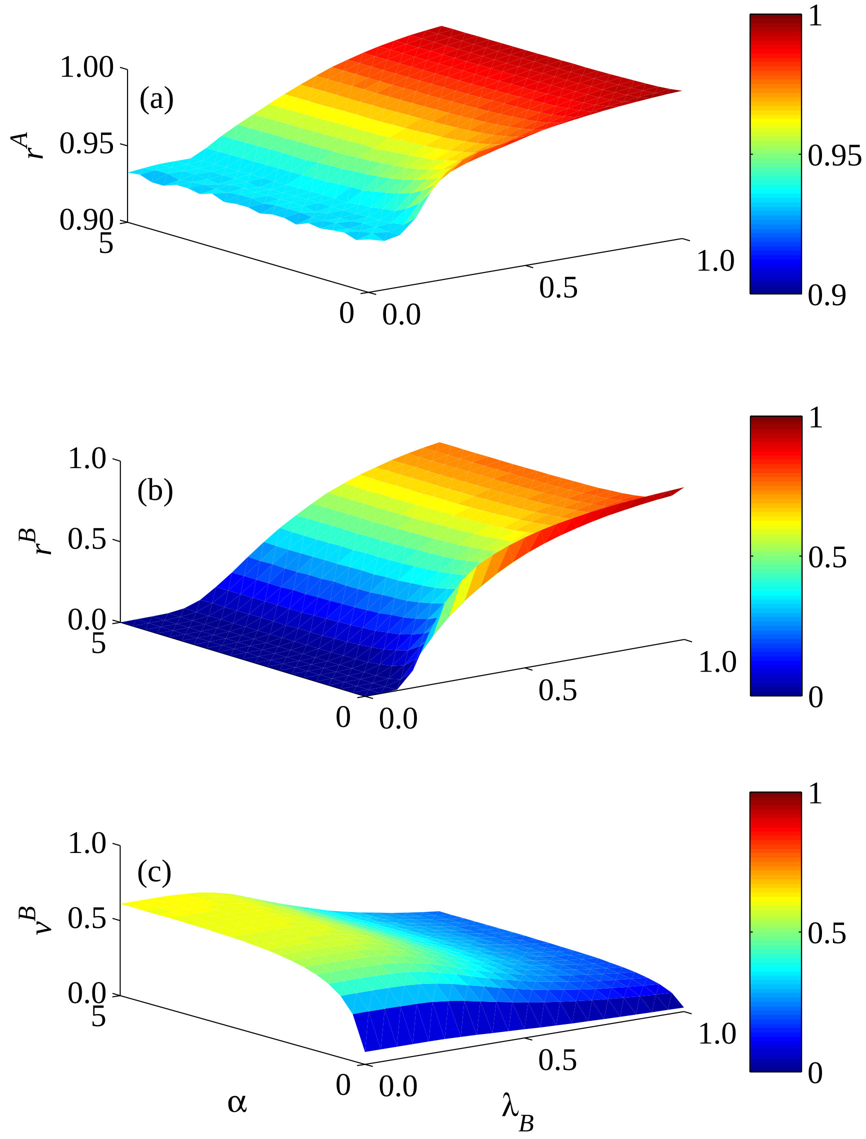

FIG. S7: **A systematic investigation of social reinforcement effect and disease transmission rate impact on final states.** For SF-SF double-layer network, (a) recovered density r^A, (b) recovered density r^B, (c) the vaccination density v^B versus α and β_B for $\lambda_A = 0.5$.

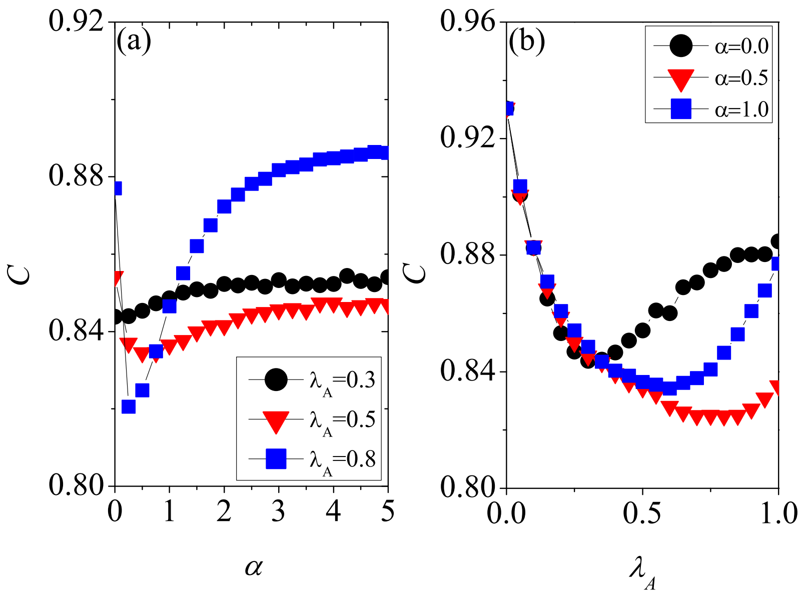

FIG. S8: **Impacts of social reinforcement effect and information transmission rate on the social cost and the optimal control.** For SF-SF double-layer network, the social cost C versus the parameters of α and λ_A in subfigures (a) and (b), respectively. Here the value of λ_B is fixed at 0.5. In (a), we select three different values of λ_A(0.3, 0.5, and 0.8), corresponding to the black circle solid, red triangle solid, and blue square solid lines, respectively. In (b), different values of α (0.0, 0.5 and 1.0) corresponds to the black circle solid, red triangle solid, and blue square solid lines, respectively.

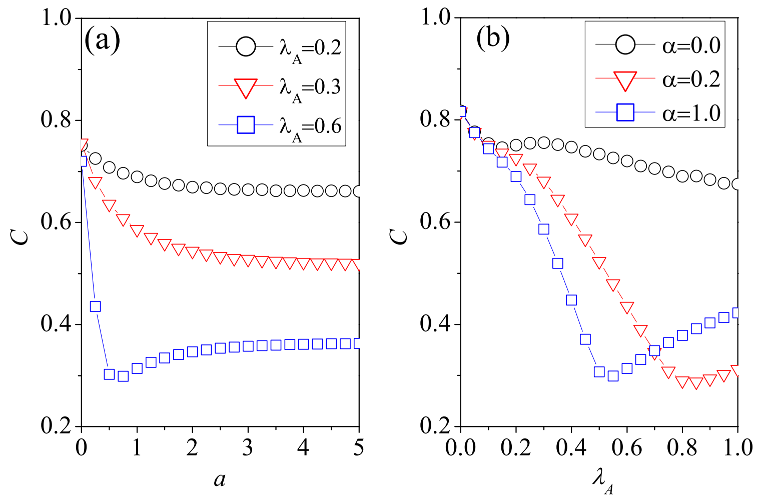

FIG. S9: **Impacts of social reinforcement effect and information transmission rate on the social cost.** For SF-ER double-layer network, the social cost C versus the parameters of α and λ_A in subfigures (a) and (b), respectively. Here the value of λ_B is fixed at 0.3. $c_R/c_V = 2$.

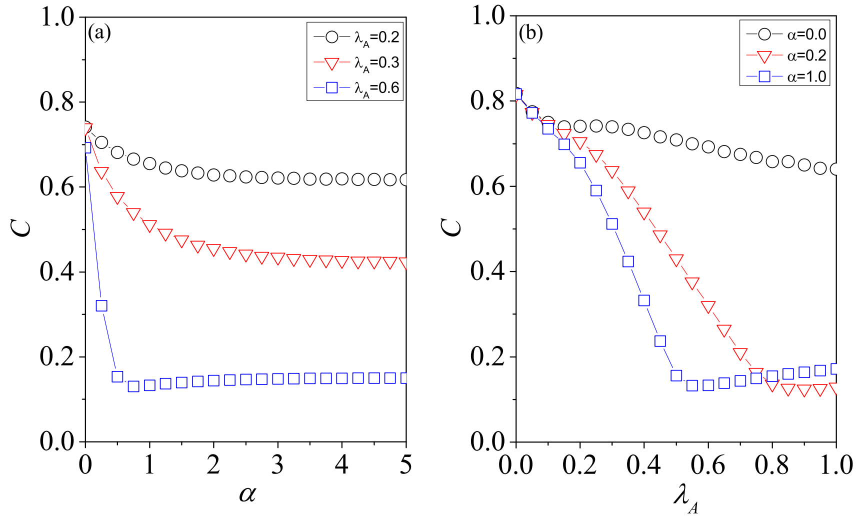

FIG. S10: **Impacts of social reinforcement effect and information transmission rate on the social cost.** For SF-ER double-layer network, the social cost C versus the parameters of α and λ_A in subfigures (a) and (b), respectively. Here the value of λ_B is fixed at 0.3. $c_R/c_V = 5$.
